# Complex Tunneling Perirectal Abscess: Intra-abdominal and Extraperitoneal Extension of a Persistent Perirectal Abscess

**DOI:** 10.7759/cureus.57688

**Published:** 2024-04-05

**Authors:** Abenezer S Tedla, Harsh R Parikh, Savni Satoskar, Jigyasha Pradhan, Shailja Kataria, Vinayak S Gowda

**Affiliations:** 1 General Surgery, BronxCare Health System, New York, USA; 2 Medicine, St. George's University School of Medicine, True Blue, GRD; 3 Surgery, BronxCare Health System, New York, USA

**Keywords:** chronic perirectal abscess, peri-rectal abscess, extraperitoneal extension of perirectal abscess, intraabdominal extension of perirectal abscess, peri-anal abscess

## Abstract

Deep-tissue extension of perianal and perirectal abscesses, while rare, requires timely diagnosis and emergent surgical intervention to prevent serious secondary complications. This report evaluates a case of intra-abdominal and extraperitoneal extension of a persistent perirectal abscess that required comprehensive irrigation, drainage, and debridement of multiple abscess-associated cavities. This report follows the case of a 24-year-old African-American female presenting to the ED with mild fevers, nausea, abdominal distension, and lower abdominal pain following a persistent perirectal abscess that had not resolved following conservative outpatient antibiotic management one week prior. Clinical examination revealed abdominal guarding with CT imaging demonstrating extraluminal air pockets in multiple intra-abdominal and extraperitoneal compartments. The patient underwent emergent surgical irrigation, drainage, and debridement of multiple abscess cavities extending from the original perirectal abscess. This report provides a comprehensive overview of the diagnosis, surgical approach, and postoperative management in a patient presenting with a complex tunneling perirectal abscess forming intra-abdominal and extraperitoneal abscesses.

## Introduction

Perianal and perirectal abscesses are common anorectal infections. The infectious etiology primarily involves an obstructed anal crypt gland providing a nest for bacterial growth and subsequent inflammation [1]. The consequent inflammatory debris and pus collect within the subcutaneous tissues and intersphincteric planes, forming abscesses, tunneling fistulae, or the habitat for chronic infections [2].

Clinically, abscesses manifest with severe episodic pain in the anal and rectal area, exasperated with activities that provoke areas adjacent to the infectious collection. Additionally, it is usually accompanied by constitutional symptoms of infection, such as fever, malaise, and fatigue [1-3]. A physical examination will frequently reveal a patch of erythematous or indurated skin overlying the abscess collection, demarcated with an area of tender fluctuance as a consequence of accumulated pus and infectious debris. On occasion, drainage of pus from the abscess can also be visualized [2,3].

Uncharacteristically, deep-spreading perianal and perirectal abscesses present with an insidious onset of clinical symptoms, often presenting only with non-specific lower abdominal pain. This both delays diagnosis and introduces anatomical complexity for definitive surgical management, increasing the risk of severe sepsis and death [2-4]. A detailed patient history, a complete physical and clinical examination of the patient, and a high degree of suspicion can assist in timely diagnosis and decisive treatment to reduce patient morbidity [2,3].

In this report, we review a case of a perirectal abscess with deep extension into the intra-abdominal, extraperitoneal, and pelvic compartments, discovered on exploratory laparotomy in a patient with suspected rectal perforation secondary to persistent perirectal abscess and clinical signs of worsening sepsis.

## Case presentation

This is the case of a 24-year-old African-American female, JR, with a past medical history of attention-deficit hyperactivity disorder (ADHD), asthma, and right-sided blindness secondary to traumatic globe rupture. JR presented to the emergency department (ED) with a one-week history of progressively worsening lower abdominal pain, nausea, and anorexia. During the interview, JR stated she had been seen in an urgent care center two weeks prior for similar complaints and was identified with a self-draining perianal/perirectal abscess. Conservative management was decided, and the patient was discharged with a 10-day course of 800-160 mg trimethoprim-sulfamethoxazole without further surgical intervention.

On presentation to the ED, JR was febrile with a temperature of 100.4°F, hypotensive with a blood pressure of 103/65 mmHg, and in generalized discomfort. Examination of the abdomen revealed diffuse distention of the lower abdomen with localized tenderness and guarding of the left lower quadrant. Further examination elicited a draining perirectal abscess with minimal pus. Complete blood count (CBC) identified an elevated white blood cell (WBC) count at 27,400, with neutrophilic shift (96.2%). A comprehensive metabolic panel (CMP) highlighted mild electrolyte derangements: hyponatremia = 129 mEq/L; hyperkalemia = 5.3 mEq/L. Additional preliminary blood tests revealed an elevated lactic acid (2.9 mmol/L). This composition of patient history, clinical symptoms, and laboratory results indicated early signs of sepsis secondary to persistent perirectal abscess. The patient was referred for CT imaging of the abdomen and pelvis with IV contrast due to concerns for abdominal guarding on physical examination and visualization of perirectal abscess depth.

**Figure 1:**
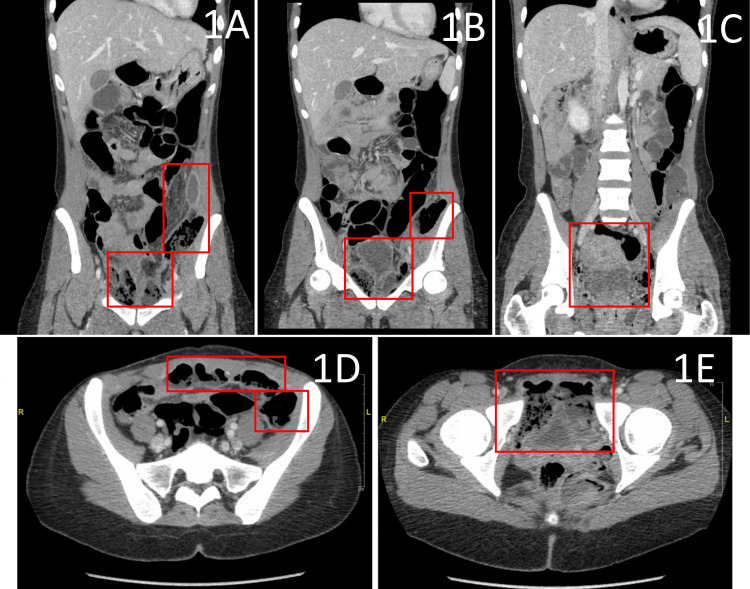
CT imaging of the abdomen and pelvis with IV contrast. Coronal images demonstrate extraluminal air and abscess pockets in the left-sided perineum (A-C), with extension along the left lateral pelvic reflection (B), indicated by the red boxes. Axial CT imaging evidences abscess extension into the anterior intra-abdominal wall (D) extending from the underlying perineal abscess cavity (E), also indicated in red. Extraluminal air pockets and possible stool were initially interpreted as concerns of bowel perforation, providing an indication for emergent surgical intervention.

CT imaging revealed significant extraluminal air and stool within the lower abdomen and pelvis, starting below the umbilicus within the lower left perineum (Figure 1A) and extending along the left lateral pelvic reflection (Figures 1B, 1C), involving both the anterior intra-abdominal wall (Figure 1D) and pelvic floor (Figure 1E). The CT was reported as concerning for bowel perforation with leakage of stool and air into the left hemipelvis. The patient was referred for emergent exploratory laparotomy and surgical exploration of the lower abdomen, incision and drainage (I&D) of the perirectal and deep intra-abdominal abscesses, and rigid diagnostic proctosigmoidoscopy.

The abdominal cavity was accessed via a midline incision extending from the umbilicus down to the pubis. A large abscess-associated cavity was identified in the anterior abdominal wall, deep to the fascia but anterior to the peritoneal cavity. An estimated 350cc of foul-smelling purulent and necrotic fluid was drained. Upon drainage of the abscess fluid, the abscess-cavity was tracked to be extending in both directions of the pelvis extraperitoneally.

On the left, the cavity extended posteriorly along the lateral perineal reflection. Multiple loculations were broken with blunt finger dissection and the cavity was continuously irrigated with normal saline and hydrogen peroxide and drained for purulent fluid. Upon dissection of loculations and evacuation of necrotic fluid, it was noted that the left-sided cavity also extended antero-caudally toward mons pubis with a small sinus draining purulent discharge into the left perineum. The sinus was expanded with a 4-cm incision to access the abscess cavity extending into the perineum. Additional necrotic loculations were broken down with blunt finger dissection with continuous irrigation and suction of the perineal abscess cavity. Necrotic muscle was also visualized, excised, and sent for culture and sensitivity.

On drainage of purulent fluid, the abscess was visualized to now extend posteriorly toward the rectum. Then, returning anteriorly to the intra-abdominal-pelvic abscess cavity, along the superior reflection of the perineum. At this point, a diagnostic rigid proctosigmoidoscopy was performed to evaluate for bowel perforation, as was indicated in preoperative imaging. On extending the sigmoidoscope 25 cm, no mucosal abnormality was noted in the rectum or distal sigmoid colon, suggesting no evidence of perforation or defects. The sigmoidoscope was removed and attention was diverted back to irrigation & drainage of remaining abscess-associated cavities.

All remaining cavities were thoroughly irrigated and the remaining purulent fluid was drained. Subsequently, a pulse irrigator was utilized for final repeat irrigation utilizing 4 L of warmed saline to wash all abscess-associated cavities in the intra-abdominal wall, extraperitoneal extensions, and perineum.

Following final irrigation and drainage, a total of four drains were placed for subsequent postoperative drainage. First, a 26-French Malecot catheter was secured to the perineal incision, draining the left preperitoneal area underlying the initial anterior intra-abdominal abscess cavity. Second, a 0.5-inch Penrose drain was secured to the perineal incision, extending posteriorly toward the rectum and presacral area. Third, a 0.5-inch Penrose drain was inserted through a stab incision draining the left perineum. Fourth, a 19-French Blake drain, connected to a suction bulb, was inserted into the right preperitoneal plane, exiting to the right of the initial anterior intra-abdominal abscess cavity. All drains were secured via 2-0 silk sutures. Midline fascia was primarily closed with interrupted 0 PDS (polydioxanone) sutures and skin loosely approximated with three skin staples and sterile dressing.

In summary, JR experienced a nine-day hospital course, receiving surgical I&D of intra-abdominal and extraperitoneal abscesses, extending from a persistent perirectal abscess. The patient was discharged on postoperative day nine following an uneventful postoperative hospital course, receiving daily wound care and IV antibiotics: piperacillin-tazobactam (Zosyn) and metronidazole (Flagyl). Three of the four drains were removed prior to discharge; the flank rectal-presacral 0.5-inch Penrose drain was left in place due to continued purulent drainage with plans for removal during outpatient clinic follow-up. On discharge, the patient was ambulating with assistance from a rolling walker, was afebrile, and had a normalized WBC count of 8,700. The patient was discharged with a seven-day course of 875-125 mg amoxicillin-clavulanate (Augmentin) and a clinic follow-up appointment on postoperative day 17. At the outpatient clinic follow-up, the final drain was removed and no further complications were noted on examination.

## Discussion

Perianal and perirectal abscesses are amongst the most common colorectal diseases encountered in the ED [1,3]. In the United States, incidence is estimated at 70,000-90,000 cases annually [2]. They represent the infectious consequence of an obstructed anal crypt gland [1]. Perianal abscesses are characterized as simple anorectal abscesses without extension into the deeper planes of the anorectal tissues [1,4]. In contrast, perirectal abscesses are more complex, with the involvement of deeper tissues [1]. A perirectal abscess can be classified as either ischiorectal, intersphincteric, supralevator, or horseshoe abscess, dependent on the involvement of the relevant deeper planes of the anorectal tissue [1,3,5]. However, perirectal abscesses also present the opportunity for cephalad abscess extension into the various intra-abdominal and extraperitoneal compartments, introducing anatomical complexity and complicating the surgical approach in definitive I&D treatment [5,6]. Furthermore, perirectal abscess extension into the deeper tissue also introduces the potential for seeding and compromise of nearby organ structures, increasing the potential for severe complications: bowel or bladder rupture, intra-abdominal tunneling and chronic fistulae formation, and infectious seeding of adjacent tissue with consequent sepsis [1,5-7]. As seen in this case, an undermanaged perirectal abscess led to cephalad and circumferential abscess extension into the perineum, intra-abdominal pelvic walls, and multiple extraperitoneal compartments.

In contrast to simple and acutely presenting perianal/perirectal abscesses, deep-spreading abscesses present with a more insidious and non-specific clinical presentation [2-4]. The rarity of the condition and its non-specific presentation provides a distinct diagnostic challenge for physicians [5]. Therefore, a detailed patient history, a complete physical and clinical examination, a high degree of clinical suspicion in light of extenuating clinical factors, and diagnostic imaging can prove invaluable for timely diagnosis and definitive surgical management prior to the onset of serious complications [3-6]. In the case of patient JR, clinical symptoms were limited to non-specific signs of fever, malaise, abdominal distension, and lower abdominal pain. However, abdominal guarding on physical examination, recent clinical history of conservatively managed perianal/perirectal abscess, and diagnostic CT imaging revealing extraluminal air provided clinical indications for deep abscess extension requiring emergent surgical exploration, irrigation, and debridement.

The primary treatment strategy for either perianal or perirectal abscess involves surgical I&D [1,3,5,6]. The procedure is traditionally performed at the bedside, with a follow-up short course of empiric antibiotics to prevent systemic infectious spread [2,5,6]. In scenarios involving either intra-abdominal or extraperitoneal extension of perianal/perirectal abscesses, the gold standard involves open irrigation, drainage, and debridement of abscess cavities with follow-up cultures and targeted pathogen-specific antibiotics in the postoperative period [2,5,6]. On occasion, fistulotomy may also be indicated for excision of abscess-associated chronic fistulae and prevention of recurrent abscess formation [2,6]. In the case of abscess extension with massive preperitoneal or retroperitoneal compartment involvement, direct access to the peritoneal cavity is avoided due to the significant risk of deeper intra-abdominal contamination and secondary peritonitis [2,7]. In these circumstances, either multiple abdominal stab-like incisions or midline abdominal incisions for extraperitoneal access have been reported with good outcomes [2,5-7]. Either surgical technique allows for appropriate access to the intra-abdominal abscess cavities, providing maneuverability and visibility for appropriate dissection, debridement, irrigation, and drainage of deep-penetrating abscesses [2,5-7]. Following the appropriate surgical evacuation of abscess cavities, wounds are primarily closed with either multiple drains or negative-pressure vacuum-assisted devices [5-7]. In our case, JR was treated with an open I&D utilizing a lower midline abdominal incision, which concluded with the placement of four independent drains at points of maximal drainage during the procedure.

## Conclusions

Perianal and perirectal abscesses are among the most common colorectal diseases presenting to the ED. The majority of cases are simple and can be managed acutely with bedside I&D procedure, followed by a short course of empirical antibiotics. However, in rare circumstances, the abscess may extend into the deeper intra-abdominal and extraperitoneal compartments. Deep extension of perirectal abscesses presents insidiously with non-specific clinical signs and requires a high degree of clinical suspicion in light supporting clinical evidence for prompt diagnosis and timely definitive surgical intervention.
